# Synthetic Vulnerabilities in the KRAS Pathway

**DOI:** 10.3390/cancers14122837

**Published:** 2022-06-08

**Authors:** Marta Roman, Elizabeth Hwang, E. Alejandro Sweet-Cordero

**Affiliations:** Department of Pediatrics, University of California San Francisco, San Francisco, CA 94158, USA; marta.romanmoreno@ucsf.edu (M.R.); elizabeth.hwang@ucsf.edu (E.H.)

**Keywords:** KRAS, cancer, synthetic lethality

## Abstract

**Simple Summary:**

Despite recent dramatic progress, developing drugs that target oncogenic *KRAS* or its key effectors remains a major challenge for cancer research. Improving our understanding of the underlying biology of *KRAS* in cancer will identify potential codependent vulnerabilities or synthetic lethal partners that are essential specifically in the context of *KRAS* mutations. Aberrant alterations in the *KRAS* oncogene not only favor cancer cell survival and proliferation, but also trigger oncogenic stress and compensatory mechanisms in cancer cells. These effectors are, thus, rational targets for defining synthetic lethal approaches to form the basis for effective therapies directed at *KRAS*-mutant tumors.

**Abstract:**

Mutations in *Kristen Rat Sarcoma* viral oncogene (*KRAS)* are among the most frequent gain-of-function genetic alterations in human cancer. Most *KRAS*-driven cancers depend on its sustained expression and signaling. Despite spectacular recent success in the development of inhibitors targeting specific *KRAS* alleles, the discovery and utilization of effective directed therapies for *KRAS*-mutant cancers remains a major unmet need. One potential approach is the identification of *KRAS*-specific synthetic lethal vulnerabilities. For example, while *KRAS*-driven oncogenesis requires the activation of a number of signaling pathways, it also triggers stress response pathways in cancer cells that could potentially be targeted for therapeutic benefit. This review will discuss how the latest advances in functional genomics and the development of more refined models have demonstrated the existence of molecular pathways that can be exploited to uncover synthetic lethal interactions with a promising future as potential clinical treatments in *KRAS*-mutant cancers.

## 1. Introduction

Since the initial identification of mutations in human *Rat Sarcoma* virus *(RAS)* genes in 1982, significant interest has focused on Ras structure and biology [1]. Ras proteins belong to the small GTPase protein family and consist of four members encoded by three genes (*KRAS4a, KRAS4b, HRAS* and *NRAS*) that share high sequence homology with the exception of the C-terminal hypervariable region [2]. Ras proteins cycle between active guanosine-5′-triphosphate (GTP)- and inactive guanosine diphosphate (GDP)-bound conformations with the aid of regulatory guanine nucleotide exchange factors (GEFs) and GTPase activating proteins (GAPs). Oncogenic mutations attenuate both the intrinsic GTP hydrolysis and GAP-stimulated GTP hydrolysis of Ras proteins, increasing the GTP-bound active state and resulting in persistent binding to a spectrum of downstream effectors, which include more than 20 different proteins from 10 effector families [3,4]. Oncogenic mutations in *RAS* genes occur in approximately 30% of all human cancers (most commonly in lung, colon and pancreatic cancer), with *KRAS* mutations alone representing 85% of all *RAS*-driven cancers [5]. The majority of oncogenic *RAS* mutations are observed in codons 12, 13 and 61, and each of these are associated with differences in patient survival, downstream signaling outputs and oncogenic potential [6]. Within this broad category of *RAS* driver mutations, it is well established that observed frequencies of a given isoform, codon site and amino acid alteration are stereotyped by histological type, as reviewed extensively in Moore et al. [7]. In addition, *RAS* mutations have been implicated in promoting cellular transformation through a wide spectrum of mechanisms beyond the canonical mitogen-activated protein kinase (MAPK) cascade, such as autophagy [8], metabolic reprogramming [9], apoptotic evasion [10] and genomic instability [11], several of which have resulted in therapeutic candidates. Importantly, it has been widely demonstrated that cancer cells harboring oncogenic mutations in *KRAS* are frequently dependent on continued activation and signaling to proliferate and survive, both in vitro and in vivo. This phenomenon is known as “KRAS addiction” and poses KRAS, together with its effectors, as appealing targets for therapeutic intervention [12,13,14,15,16,17].

## 2. Strategies to Target Oncogenic Mutant KRAS

### 2.1. Direct KRAS Inhibition

To date, numerous approaches to directly target KRAS [18,19,20,21] and its post-translational modifications, which promote the association of KRAS to the cell membrane, have been investigated [22,23,24]. The discovery in 2013 by Ostrem et al. of a covalent inhibitor to lock the GDP-inactive form of KRAS G12C marked the beginning of a new era in the development of KRAS inhibitors [20]. Subsequently, other research groups reported the discovery of small molecules with similar mechanisms but improved binding and pharmacologic properties. ARS-853 [18,21] was the first direct KRAS G12C inhibitor that proved both efficacious and selective in *KRAS*-dependent cells. In 2018, Janes et al. [25] reported a new and improved KRAS G12C inhibitor (ARS-1620) that overcame the limitation of the previous compounds regarding their use in in vivo models, demonstrating that targeting switch II of KRAS G12C is a viable and promising clinical therapeutic strategy. One of these covalent inhibitors of KRAS G12C (sotorasib) was clinically approved in 2021 for the treatment of advanced non-small cell lung cancer (NSCLC) patients carrying a G12C mutation in the *KRAS* oncogene [26,27,28,29]. Ongoing clinical trials are evaluating the toxicities and efficacies of monotherapies as well as therapeutic combinations in eligible patient populations [30,31]. A multicenter phase 1 trial of AMG 510 (sotorasib) was performed in patients with advanced solid tumors harboring the G12C mutation (*n* = 129; including NSCLC and colorectal cancer patients, among others). This study demonstrated a durable clinical benefit from sotorasib with relatively low toxicity in a heavily pretreated patient cohort: most strikingly, 32.2% of NSCLC patients had a confirmed response and a majority (88.1%) stable disease. The results for non-lung cancer patients were less promising. Three of 42 patients with colorectal cancer (7.1%) showed a partial response and 66.7% disease control, reinforcing the contribution of disease heterogeneity to driver mutation susceptibility [32]. A remaining barrier is the genotype specificity of current clinically available inhibitors, as G12C mutations account for nearly half (46%) of mutations in NSCLC [7] but only 4–14% of KRAS mutations in all human cancers [33]. One of the unique features of the *KRAS* G12C mutant is its retention of near wild type intrinsic GTPase activity, allowing covalent inhibitors to selectively target the inactive GDP state and retain high efficacy [34]. In contrast, the *KRAS* G12D mutant demonstrates a high nucleotide exchange rate. Thus, new strategies are being explored to discover inhibitors of both the GDP and GTP-bound states, such as non-covalent inhibition of the switch II region outside of the nucleotide-binding site. Other approaches that may be more generalizable to multiple alleles include PROTAC protein degraders targeting KRAS [35] or its signaling partners, as well as steric targeting of effector engagement by Ras-GTP [36].

Clinical trials for NSCLC patients with *KRAS* non-G12C mutations have previously been reviewed [37]. The long-term efficacy of the current *KRAS* G12C inhibitors remains unclear, mostly due to toxicity and acquired resistance mechanisms [26,29,38,39]. Notably, nearly all patients included in early-phase, single agent clinical trials of both sotorasib and adagrasib (MRTX949) developed acquired resistance and demonstrated disease progression despite the initial response. Next-generation sequencing analysis of adagrasib-resistant tumors suggested that the majority (45%) of identifiable mechanisms occurred as either secondary alterations in either *KRAS* itself, including within the targeted switch II pocket, or in members of the receptor tyrosine kinase (RTK)-RAS-MAPK pathway, emphasizing its importance as a major mechanism of survival and proliferation in this tumor context [40]. This fact highlights the continued need to identify and develop combinatorial therapies [39,41,42] or explore alternative opportunities for targeting *KRAS*-driven cancer.

### 2.2. Indirect KRAS Inhibition

Indirect strategies for targeting the KRAS pathway can be classified in two main groups: (1) inhibition of upstream KRAS activators and (2) inhibition of downstream KRAS canonical effectors.

With regards to upstream KRAS activation, most studies have focused on blocking KRAS upstream RTK signaling through the epidermal growth factor receptor (EGFR) and other RTKs. However, clinical studies have indicated that patients harboring *KRAS*-mutant tumors are not sensitive to EGFR tyrosine kinase inhibitors (TKIs). In fact, KRAS activation is one of the signaling pathways conferring resistance to EGFR TKIs [43,44]. Although it has been demonstrated that the deletion of *EGFR* transiently reduces *KRAS*-mutant tumor growth, EGFR therapies trigger tumor escape mechanisms involving non-EGFR ERBB family members [45]. During the last few years, significant interest has focused on the protein tyrosine phosphatase SHP2. SHP2 acts downstream of many RTKs and mediates cellular signaling through the RAS/MAP kinase pathway. Several studies have provided evidence for a critical dependence of mutant *KRAS* on SHP2 and have shown the potential clinical use of combined SHP2/MEK inhibition for *KRAS*-driven tumors [46,47]. Two promising inhibitors of SHP2 (RMC-4630 and TNO155) are currently undergoing clinical trials [48,49]. Other approaches rely on blocking SOS1, a relevant GEF for KRAS, and suggest efficiency in combination with MEK inhibition [50,51] in the context of *KRAS* mutations.

**Figure 1:**
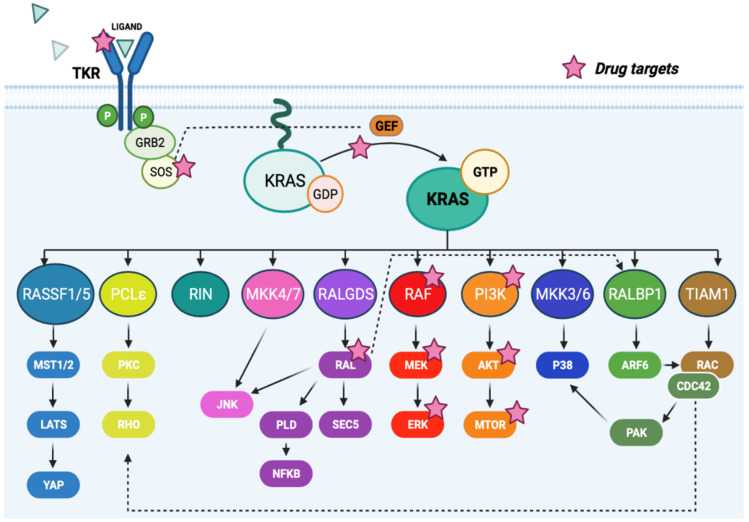
KRAS downstream effector pathways. The active form of KRAS (KRAS-GTP) regulates many signaling pathways affecting essential cellular functions such as cell proliferation, migration, survival, differentiation, endocytosis, migration and angiogenesis through the interaction with different effectors. Adapted from Soriano et al. [52]. Further detail regarding therapeutic approaches is provided in Table 1. Figure made in https://biorender.com (accessed on 31 May 2022).

The therapeutic potential of inhibiting downstream KRAS effectors (Figure 1) has been the focus of intensive investigation. Table 1 summarizes some of the most relevant inhibitors targeting the RAS pathway.

While many KRAS effector pathways have been described, the most well-studied is the MAPK cascade, which regulates tumor cell proliferation and survival [68,69]. A number of inhibitors targeting the MAPK pathway have been developed and tested as single agents or in combination with chemotherapy in different *KRAS*-driven cancers in the clinic [70,71,72]. The limited efficacy of these inhibitors is likely explained by the rapid development of multiple feedback mechanisms that are able to re-activate the MAPK pathway at different signaling levels [73,74]. The phosphatidyl-inositol 3-kinase (PI3K) pathway is also critical in KRAS signaling, and inhibitors against its effectors are currently under clinical evaluation. However, mutant oncogenic *RAS* has been described as a dominant determinant of resistance to PI3K inhibitors even in tumors with coexisting mutations in *PI3K*, with *c-MYC* and *CYCLIN B* acting as potential mediators of such resistance [75]. Studies targeting the nuclear factor kappa B (NF-kB) pathway, activated by RAL, have demonstrated that *KRAS*-mutant tumor cells require *NF-kB* for viability [76] and inhibitors targeting this effector are also being tested in clinical trials [77]. A less characterized effector of RAL is phospholipase D (PLD), which is associated with the generation of lipid second messengers such as phosphatidic acid, lysophosphatidic acid and diacylglycerol. The activation of PLD does not depend on GDP/GTP exchange, but it needs the additional association with the GTPase ARF [78]. It has been demonstrated in the preclinical setting that targeting PLD survival signals in human cancer cells with *RAS* mutations could be an effective strategy to induce apoptosis. This node of RAS signaling portrays an opportunity for the development of novel anticancer drugs [79,80]. The therapeutic value of less studied KRAS canonical effectors, such as RIN, TIAM1 or MKK4/7, remains unknown.

In addition to emergent resistance, effector targeting is further complicated by heterogeneity in both mutation-specific affinities as well as heterogeneity of effector dependencies [7,81]. One example of this is that cell lines harboring a *KRAS* G12D mutation revealed increased sensitivity to MEK and RAF combination therapy relative to non-G12D *KRAS* mutations. This observation led the authors to hypothesize that, in the presence of MEK inhibition, mutant *KRAS* alleles with high intrinsic nucleotide exchange are dependent on RAF dimerization to maintain a GTP-bound state [82]. Currently, significant clinical barriers to complete MAPK blockade are dose-limiting toxicities as observed both in cell lines [83] and patients, and most clearly evidenced in clinical trials testing BRAFi in combination with trametinib (MEKi) in melanoma patients [84]. However, preclinical studies indicate that KRAS-targeting covalent inhibitors may synergize with upstream activators such as EGFR and IGF1R or downstream effectors such as MTOR while minimizing toxicity, suggesting that these combinations may result in more durable responses while mitigating the deleterious side effects of MAPK blockade [21,39].

In summary, studies targeting KRAS downstream signaling suggest that the inhibition of a single effector arm will be of limited efficacy due to compensatory feedback mechanisms. Thus, although the inhibition of KRAS effectors is a potential strategy to target *KRAS*-driven cancers, it remains a significant challenge, and successful targeting of *KRAS*-mutated tumors will likely require simultaneous targeting of multiple effector pathways [85,86].

### 2.3. Synthetic Lethality

**Figure 2:**
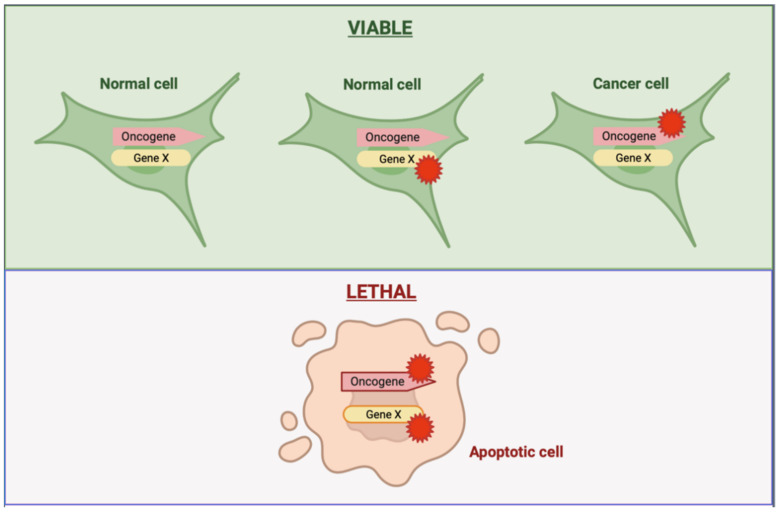
Synthetic lethality as a therapeutic strategy in cancers driven by oncogenes. Synthetic lethality happens when the alteration of an oncogene or gene X in isolation is compatible with cellular viability, whereas loss of both genes together leads to cellular lethality. Cancer-driving genetic alterations are commonly associated with dependencies that are specific to these alterations and absent in normal non-neoplastic cells. The presence of one of these dependencies in cancer cells but not in normal cells can therefore create opportunities to selectively kill cancer cells by mimicking the effect of the second genetic mutation with targeted therapy. Figure made in https://biorender.com (accessed on 31 May 2022).

A synthetic interaction occurs when the perturbation of a single gene alone is viable, but the perturbation of two genes together results in a new phenotype, most often lethality [87,88] (Figure 2). The interest in the discovery of novel pro-oncogenic partners contemporary with mutant *KRAS* has potentially increased during the last decade, an effort that has been fostered by technological advances in loss-of-function screens.

Synthetic lethality can be triggered by: (1) the loss of function of two genes belonging to the same essential signaling pathway, (2) two genes that are capable of activating the same pathway through different signaling cascades or (3) genes that act in distant pathways converging upon a specific cellular perturbation [89]. Specific genetic alterations in *KRAS*-mutant cancer cells appear to confer such vulnerabilities and can be therapeutically targeted. Many of these vulnerabilities emerge as a consequence of the adaptive process to constitutive oncogene activation and are related to the overall stress state induced by mutant *KRAS*, including DNA damage or replication stress, proteotoxic stress, mitotic stress, metabolic stress or oxidative stress, reinforcing the pleiotropic mechanisms exploited by the *KRAS* oncogene [90,91,92,93,94]. Thus, identifying essential genes and/or signaling pathways that disrupt this *KRAS*-driven cell state and impair cell viability has become the focus of intense investigation since it could result in novel and potentially less toxic therapies, as non-mutant cells would theoretically survive.

Additionally, if the targeted synthetic lethal partner is selective for a *KRAS*-specific mutation, this mutation could be used as a biomarker to stratify patients for treatment. However, the identification of potential synthetic lethal interactions is hampered by variability associated with genetic backgrounds/cellular conditions and the uniqueness of these interactions to specific cellular contexts [88,95], including cell extrinsic factors, such as the requirement for asparagine biosynthesis under nutrient-deprived conditions in a *KRAS*-dependent manner [94]. Such heterogeneity and genetic variability may explain why, to date, therapies based on synthetic lethality have not yet proved clinically effective. This fact highlights the importance of performing large-scale high-throughput synthetic lethal screening approaches, taking advantage of technological advances in RNA interference (RNAi) and CRISPR systems, with the aim of identifying essential genes and vulnerabilities in the context of oncogenic KRAS signaling. Over the last several years, various studies, whether hypothesis-driven studies, or drug-based or genetic-based screens, have brought to light novel *KRAS* synthetic lethal partners. These studies have been of great value in increasing our knowledge regarding signaling pathways required for oncogenic *KRAS* function, although their clinical value still remains unknown [96]. Here we discuss the latest advances in functional genomics and in the development of more refined models, and how these have uncovered molecular pathways through which synthetic lethality can be exploited as a potential clinical treatment in *KRAS*-mutant cancers.

## 3. Screening Approaches to Identify Synthetic Lethal Interactions with *KRAS*

The two main genetic approaches to identifying synthetic lethal targets in human cancer cells are loss-of-function screens based on RNAi and CRISPR/Cas9. The development of these tools has made possible unbiased, genome-wide studies in human cells possible. To define synthetic lethal partners in the context of *KRAS*, these screens are usually performed either on isogenic *KRAS*-mutant or *KRAS* wild type cell systems, or on wide panels of different *KRAS*-mutant or wild type cell lines. Moreover, different screening approaches have been implemented in order to identify genetic vulnerabilities for *KRAS* tumors, including arrayed formats (i.e., the effect of the loss of each gene is analyzed in individual wells) and pooled formats (i.e., where changes in the relative abundance of individual barcodes are quantified) [95,97]. These screens have confirmed the dependency of many *KRAS*-mutant cell lines upon *KRAS* itself and identified potential synthetic lethal genes in *KRAS*-driven cancers.

### 3.1. RNA Interference Screens

In RNAi screens, exogenous short interfering RNAs (siRNA) or short hairpin RNAs (shRNA) are introduced into human cells. These small RNA sequences are then loaded into the endogenous RNA-induced silencing complex (RISC), allowing the knockdown of complementary target mRNAs [98]. This tool provided the first opportunity to carry out scalable genetic screens in human cells, and many studies have reported numerous genes as synthetic lethal interactors with oncogenic *KRAS* including *PLK1, TBK1, WT1, STK33, FGFR1, YAP1* and *XPO1*, among others [76,85,99,100,101,102,103]. In fact, some ongoing clinical trials are testing the efficacy of PLK1 inhibitors, CYC140 (phase I: NCT03884829) and BI-2536 (phase II: NCT00710710), in advanced leukemias and pancreatic cancers, respectively [104]. However, despite the vast amount of knowledge these RNAi-based screens have enabled, there are several limitations, including a substantial number of off-target activities of RNAi libraries, resulting in a lack of overlap in findings between independent screens [5,95]. The inconsistencies in the experimental results between studies is thus reflected in the relatively small number of robust synthetic lethal targets that have been identified by this type of screening. Such limitations likely contribute to false-negative and false-positive rates and are attributed to the use of different RNAi libraries, the use of cell lines with different genetic backgrounds as well as the different screening modalities and quantification methodologies [105,106]. The most informative RNAi screens in the context of *RAS*-mutant cancers have been previously reviewed by Ebi et al. [107], Downward et al. [95] and Aguirre et al. [96].

### 3.2. CRISPR/Cas9 Screens

Over the last decade, CRISPR/Cas9 technology has emerged as an alternative for uncovering new synthetic lethal partners in the biology and treatment of cancer, revolutionizing the field of loss-of-function screens [108,109,110]. CRISPR/Cas9 genome editing technology uses a 20-nucleotide guide RNA (gRNA) that guides the Cas9 nuclease to a specific target site generating precise DNA double-strand breaks [111]. A number of studies have confirmed that CRISPR-based screens have improved reproducibility compared to RNAi screening approaches, likely due to the lower off-target frequency of gRNAs and the higher efficiency of CRISPR reagents from creating knockout mutants rather than RNA-targeted knockdowns [112,113,114]. Thus, large-scale CRISPR/Cas9 screens have proven to be a powerful method identifying genetic defects in tumors harboring oncogenic mutations such as *KRAS* [115,116]. Table 2 summarizes a selection of the most relevant CRISPR/Cas9 screens carried out to date, focusing on a few illustrative examples below.

Pioneering work in the use of genome-wide CRISPR/Cas9 screens to identify synthetic lethal genes in the context of oncogenic *KRAS* was published by a team led by Sabatini and colleagues [115]. The authors compared six acute myeloid leukemia (AML) cell lines with mutations in either *KRAS* or *NRAS* against six *KRAS* wild type cell lines. This study highlighted the importance of targeting specific components of the RAS pathway itself in order to impact the viability of *RAS*-dependent tumor cells. Isogenic murine Ba/F3 (*NRAS*-mutant) cell lines were used to perform a parallel and independent CRISPR screen that showed a very high degree of overlap with the screen carried out in AML cell lines. Genes involved in the maturation of RAS (such as *RCE1* and *ICMT*) and genes related to MAPK pathway signaling (*RAF1* and *SHOC2*), supported the central role of MAPK signaling in *RAS*-mutant cancers. In this study, the authors validated PREX1, a GEF for the Rac GTPases, and described it as a novel *RAS* synthetic lethality [115].

Yau et al. [118] performed an in vivo pooled human genome-wide CRISPR/Cas9 knockout screen of tumor xenografts using a well-characterized isogenic pair of human colorectal cancer cell lines harboring either mutant or wild type *KRAS*. The primary aim of this screen was to extend the knowledge of the genetic vulnerabilities of mutant *KRAS* tumors to the in vivo setting. They identified approximately 250 gene candidates that were used to design a second smaller focused in vivo screen, with higher depth and coverage per construct, to validate the genome-wide screen. Comparing *KRAS*-mutant to *KRAS* wild type cells, they found gene knockouts that conferred selectively beneficial or detrimental viability effects in the context of KRAS activation. Pathway analysis identified multiple metabolic vulnerabilities (NAD kinase and ketohexokinase), highlighting the therapeutic potential of targeting cancer metabolism, associated with the rewiring of metabolic programs that promote tumor survival, growth and immune evasion in different *KRAS*-mutant cancer types [17,133]. This work further identified *INO80 Complex Subunit C* (*INO30C*) as a novel *KRAS*-dependent tumor suppressor gene in both colorectal cancer and pancreatic adenocarcinoma isogenic xenografts.

Although many studies have demonstrated the impact of targeting single KRAS downstream effectors, the appearance of resistance and compensatory signaling mechanisms highlights the need to use combination therapies. For this reason, multiple high-throughput CRISPR screening approaches have been applied to identify critical genes that contribute to drug resistance in *KRAS*-mutant human cancers [132]. Šuštić et al. [119] identified *IRE1*, a proteotoxic stress response gene, as a vulnerability in the context of *RAS* mutations in a *RAS* synthetic lethality screen in yeast. However, in human cells, they found no difference in cell viability between the control and *ERN1* (*IRE1* mammalian ortholog) KO human cells, indicating the synthetic lethal interaction with *KRAS* is not conserved between human cells and yeast, which is surprising considering *RAS* is a highly conserved pathway. The authors of this work argue that this inconsistency between yeast and human cells could be due to the fact that yeast are missing the RAF/MEK/ERK MAPK cascade [134]. To corroborate their hypothesis, they investigated the effect of knocking *ERN1* out in cell proliferation in combination with a MEKi (selumetinib) and found increased MEKi sensitivity in ERN1 KO cells. This result encouraged them to perform a genome-wide CRISPR/Cas9 MEK inhibitor resistance screen to identify a mechanistic link between *ERN1* and the MAPK pathway using ERN1 KO LoVo colorectal cancer cells. This screen established a relationship between *ERN1* and *JUN* and highlighted the relevance of the ERN1-JNK-JUN pathway as a novel regulator of MEKi response in human *KRAS*-mutant colorectal cancer, providing a therapeutically exploitable vulnerability. Similarly, Szlachta et al. [120] described large-scale in vivo and in vitro CRISPR/Cas9 KO screens that also identified genes whose genetic deletion synergistically increased the cytotoxic effect of a MEKi (trametinib). They carried out the CRISPR screening using an sgRNA library enriched for epigenetic regulators, transcription factors and nuclear proteins, in a *KRAS*-mutant patient-derived xenograft (PDX) model of pancreatic ductal adenocarcinoma. This study identified multiple genes, such as *CENPE*, whose depletion creates a synthetic lethality in combination with MEK inhibition. They complemented this work by demonstrating that overall drug responses could be modeled using the DREBIC approach, which captures the relative essentiality of the drug target (gene specific CRISPR viability scores) and their basal expression levels (mRNA) for specific cell types.

In another report on MEKi synthetic lethalities, Sulahian et al. [122] performed a genome-scale CRISPR/Cas9 screen in the presence of trametinib that identified *SHOC2* as a synthetic lethality when combined with MEK inhibition in *KRAS*-mutant lung and pancreas cancers. *SHOC2* is a positive regulator of *RAF1*-mediated MAPK signaling. This work demonstrated that *SHOC2* loss conferred a consistent attenuation of MAPK pathway re-activation in response to trametinib. These data further validated results described by Wang et al. [115], where *SHOC2* was essential for proliferation specifically in *RAS*-mutant leukemia cells. Another example of combinatorial CRISPR/Cas9 and MEKi screening is the work recently published by Yun et al. [132]. Here the authors focused on *KRAS*-mutant colorectal cancer and found the RTK pathway was a resistance driver to MEK inhibitors. They showed that a combinatorial inhibition of the RTKs-GRB7-PLK1 axis and MEK could be a promising strategy in the context of *KRAS* tumors. Taken together, these studies provide support for novel treatment combinations for refractory *KRAS*-driven tumors.

CRISPR/Cas9 loss-of-function screens have become a very useful and valuable tool for identifying synthetic lethal genes that do not cooperate just with MEK inhibitors and other therapies. For example, recent work described a genome-wide CRISPR/Cas9 screen performed in both 2D and 3D conditions [123]. The aim of this work was to identify synthetic lethal targets for *KRAS*-driven lung adenocarcinoma tumors, as well as synthetic vulnerabilities in combination with a KRAS inhibitor to combat the resistance mechanisms associated with these drugs [38,39,135,136]. While 2D in vitro models have been broadly used to investigate cancer biology and drug sensitivity, 2D cultured cells are unable to truly reproduce the natural proliferation, migration, drug response and/or rewired metabolism taking place in the complex 3D environment [137,138,139] of a tumor. To overcome some of these limitations, 3D cancer cell culture systems are a valuable resource that may provide a more accurate and relevant preclinical testing model. Nevertheless, 3D models have not been widely used to perform CRISPR screening because they are much less scalable [123]. Han et al. developed a scalable method to propagate *KRAS*-mutant lung cancer spheroids that allowed them to carry out a genome-wide CRISPR screen in 3D conditions. They found a module composed of genes correlated with *carboxypeptidase D* (*CPD*) was significantly depleted in the 3D versus 2D phenotype and showed a strong synthetic lethality with the KRAS inhibitor in 3D, suggesting that CDP and its interactors could be potential therapeutic targets.

Other studies have tried different combinatorial strategies to find synthetic lethal interactors using CRISPR/Cas9 screening. For example, the transcription regulator *PRMT5* (*protein arginine methyltransferase 5*) was identified as a potential gene target in combination with gemcitabine for pancreatic ductal adenocarcinoma [129]. The novel combination of the epigenetic gene *ASF1*, a histone H3-H4 chaperone, and anti-PD1 immunotherapy for *KRAS*-mutant lung adenocarcinoma patients has been described by Li at al. after performing a custom epigenetic-focused CRISPR/Cas9 in vivo screen using a KRAS^G12C^/Trp53^−/−^ mouse model [126]. For colorectal cancer patients harboring *KRAS* mutations, 2D genome-wide CRISPR/Cas9 screens have been performed to investigate the genes required for sensitivity to the BCL/X_L_ inhibitor (ABT-263, an anti-apoptotic protein), finding multiple regulators of the WNT signaling pathway as potential synthetic lethal targets [130]. Finally, it is important to mention the use of multiomic approaches to uncover synthetic lethal combinations in the context of *KRAS.* Our research group participated in a collaborative effort to perform a CRISPR dual knockout library targeting 119 *RAS*-related genes (previously identified in an affinity purification mass spectrometry study to construct a protein-protein interaction map of RAS interactors). This approach found a number of novel lethal genetic interactions, highlighting a potent *KRAS*-dependent interaction between *RHOA* and *RAP1GDS1* genes [125]. All these studies demonstrate how CRISPR screen technologies are revolutionizing cancer research by bringing to light the molecular mechanisms of tumorigenesis and feedback mechanisms associated with treatment resistance.

As described above, high-throughput CRISPR/Cas9 screens can be used to identify potent combination therapies. However, even in the context of initially promising combination therapies, the appearance of emergency compensatory mechanisms is inevitable in most patients. To better understand this scenario, Anderson et al. designed a custom CRISPR/Cas9 library to map the landscape of druggable pathways cooperating with inhibitors of the key KRAS effectors (MEK, ERK and PI3K) in different types of *KRAS*-driven cell line cancer models [86]. They found that *KRAS*-mutant tumors are able to rapidly acquire resistance to potent combination therapies and identified strategies to potentially combat such resistance using sensitivities common between multiple models. These findings provide a starting point for the design of next-generation treatment strategies and raise hopes of finding an effective therapy for patients with mutations in the *KRAS* oncogene.

## 4. The Future of the Search for Synthetic Lethal Interactors for *KRAS*-Driven Tumors

During the last several years, the use of biocomputational methods in combination with CRISPR/Cas9 knockout screens has become a highly powerful technique to elucidate essential cancer genes and define important therapeutic targets [116,140,141,142]. Although many studies have enumerated interesting *KRAS* synthetic lethal targets, these findings are not applicable to all *KRAS*-mutant cancer contexts because of the heterogeneity associated to this oncogenic mutation. The Dependency Map (DepMap) portal [143] from the Broad Institute was created to facilitate the discovery of novel cancer vulnerabilities by providing open access to key cancer dependencies’ analytical and visualization tools. The DepMap portal integrates the Achilles Project (an ongoing systematic effort aimed at screening more than 2000 cancer cell lines of a variety of lineages in the next years), cell line database (CCLE) and drug susceptibility databases (PRISM) [144]. Genome-scale RNAi and CRISPR/Cas9 technologies were used to silence or knockout individual genes, and those affecting cell viability and survival were identified and systematically catalogued. Methods such as DEMETER2 for RNAi screening [145] and CERES for CRISPR screening [146] have been developed to computationally infer and subtract seed effects that arise for each individual gene. The information extracted from these analyses allows investigators to establish links between genetic dependencies and the genetic or molecular features of the tumors. Remarkably, the Achilles Consortium has screened over 60 different *KRAS*-mutant cell lines. Additionally, the Wellcome Sanger Institute is developing its own Cancer Dependency Map through Project Score, using genome-scale CRISPR/Cas9 screening to identify dependencies across a diverse collection of human cancer cells [147,148]. Another relevant resource for investigating context-specific synthetic lethalities is PICKLES, an integration of multiple CRISPR knockout library results. PICKLES allows the user to refer to the co-essentiality of a pair of genes, providing the possibility of studying potential combinatorial strategies [149]. Despite all these efforts to gain insights into *KRAS* basic biology and develop more effective targeted therapies for *KRAS*-mutant cancer patients, a universal synthetic lethal target across all *KRAS*-mutant contexts has not been found. This fact can be explained mainly due to the variability in *KRAS* dependency according to the cancer type, the differences in downstream signaling across *KRAS*-specific mutations and the diversity of co-occurring mutational landscapes for each cancer type [96]. This scenario highlights the importance of better understanding the different *KRAS* vulnerabilities in their specific molecular and genetic contexts.

## 5. Conclusions

Intensive efforts to identify effective therapies for patients harboring mutations in *KRAS* have produced an enormous amount of new data and knowledge about the biology of this oncogene and its effector pathways. As reviewed here, many different strategies have been tested to inhibit the consequences of oncogenic *KRAS*. The genetic concept of “synthetic lethality” is simple but continues to exert a major impact on cancer research. The growing appearance of new screening technologies and methodologies is having a significant impact in cancer biology, paving the way for new research directions. Specifically, the advances in CRISPR systems and their combination with biocomputational analyses are identifying interesting vulnerabilities and dependencies for *KRAS*-driven tumors. Nonetheless, these vulnerabilities cannot be extrapolated to all *KRAS* contexts, and it is necessary to continue exploring KRAS-related pathways within a specific tumor context to understand their implications for cancer initiation, progression and therapy. Finally, synthetic lethal screening will likely become a very useful tool in the context of the increasing usage of the KRAS direct inhibitors. Despite the high clinical impact these KRASi are demonstrating in patients harboring *KRAS* G12C mutations, untreated patients with different *KRAS*-driven cancers require greater efficacy than that seen to date with *KRAS* G12C inhibitor monotherapy. For this reason, there is a strong emphasis towards the development of combination therapies, and synthetic lethal screens could be the key to identify specific vulnerabilities along with the blockade of KRAS.

## Figures and Tables

**Table 1:** Ras pathway targeting drugs at different stages of development. Adapted from Healy et al. [53].

Drug	Target	Mode of Action	Development Stage
Cetuximab (monoclonal antibody) [54]	EGFR	Competitive inhibitor of EGFR (extracellular domain)	FDA-approved
Gilteritinib (small molecule) [55]	FLT3, AXL	Binds active FLT3	FDA-approved
BAY293 (small molecule) [50]	SOS1	Disruption of the KRAS-SOS1 interaction	Preclinical
BI1701963 (small molecule) [51]	SOS1	Prevents KRAS-SOS1 interaction binding catalytic site	Phase I (NCT04111458)
Dabrafenib (small molecule) [56]	BRAF (wt and V600)	ATP-competitive inhibitor of BRAF	FDA-approved
Vemurafenib (small molecule) [57]	BRAF V600E	ATP-competitive inhibitor of BRAF	FDA-approved
Cobimetinib (small molecule) [58,59]	RAF, MEK	Non-ATP-competitive inhibitor of active MEK	FDA-approved
Trametinib (small molecule) [58,60]	MEK	ATP non-competitive kinase inhibitor. Reduces MEK phosphorylation	FDA-approved
LY3214996 (small molecule) [61,62]	ERK	ATP-competitive inhibitor of ERK1/2	Phase I (NCT02857270)
RBC8 (small molecule) [63]	RAL	Non-ATP-competitive inhibitor of RAL-GDP	Preclinical
Alpelisib (small molecule) [64,65]	PI3Kα	ATP-competitive inhibitor of PI3Kα	FDA-approved
Uprosertib (small molecule) [66]	AKT	ATP-competitive inhibitor of AKT	Phase II (NCT01902173)
Everolimus (small molecule) [67]	mTOR	Inhibits mTOR activation after complexing with FKBP12	FDA-approved

**Table 2:** KRAS synthetic lethal CRISPR-based functional screens.

Reference	CRISPR Library	Type of Study	Cell Lines	Combined Screen	Synthetic Lethal Hits or Pathways
Wang et al. *Cell*. 2017 [115]	Genome-wide (GW) human CRISPR libraries	Pooled CRISPR-based screen (proliferation assay)	6 human KRAS/NRAS-mutant and 6 KRAS-WT leukemia cell lines	Parallel GW mouse CRISPR screen using isogenic Ba/F3 cells (NRAS)	RCE1, ICMT, RAF1, SHOC2, PREX1
Martin et al. *Cell Rep.* 2017 [117]	Genome-wide CRISPR library targeting 18,148 genes	Pooled CRISPR-based screen (proliferation assay)	Isogenic pairs of HCT116/DLD1 cells (KRAS^G13D^); LS513 cells (KRAS^G12D^). Colorectal cancer cells	shRNA library targeting 1100 essential genes	Mitochondrial protein translation, transcription and oxidative phosphorylation pathways; Mrpl52 and Ndufb10
Anderson et al. *Cell Rep.* 2017 [86]	Custom CRISPR/Cas9 library (~2000 sgRNAs) targeting 378 genes	Pooled CRISPR-based screen (drug sensitizer screening)	Pan-cancer panel of KRAS-mutant cell lines	Drug inhibition of KRAS pathways: MEK, ERK, PI3K.	MAPK14, MDM4, SRC
Yau et al. *Cancer Res.* 2017 [118]	Human GeGKO v2 library pooled plasmid (lentiCRISPRv2)	Pooled CRISPR-based in vivo screen (xenograft model)	Isogenic pairs of HCT116 cells (KRAS^G13D^). Colorectal cancer cells	Secondary smaller focused CRISPR screen targeting ~320 KRAS-related genes	NADK, KHK, SUCLA2, INO80C. Nucleotide synthesis, redox balance and mitochondrial processes
Šuštić et al. *Genome Med.* 2018 [119]	Human GeGKO v2 library pooled plasmid (lentiCRISPRv2)	Pooled CRISPR/Cas9 MEK inhibitor resistance screen	ERN1^KO^ LoVo cells (KRAS^G13D^). Colorectal cancer cells	MEK inhibitors: Selumetinib and Trametinib	DUSP4, STK40, RUNX2, CBFB, DET1, COP1. Negative regulation of the JUN signaling
Szlachta et al. *Nat Commun.* 2018 [120]	CRISPR library from Dr. Sabatini (~4000 human genes)	Pooled CRISPR knockout sensitizer screen	PDX366 model (KRAS, P53 and SMAD4 mutant). Pancreatic PDX-cells	MEKi: Trametinib	CENPE, RRM1
Dompe et al. *PLoS ONE.* 2018 [121]	Custom druggable genome CRISPR library (2194 genes)	Pooled CRISPR knockout sensitizer screen	MOR lung cancer cell line (KRAS-mutant)	MEKi: Cobimetinib and ERKi: GDG-0994. +Validation focused screens (4 KRAS-mutant lung cancer cells)	MAPK7
Sulahian et al. *Cell Rep.* 2019 [122]	Genome scale Avana-4 barcoded CRISPR library (74,687 sgRNAs)	Pooled CRISPR-Cas9 screens MEK sensitizer screen	KRAS-mutant cancer cell lines (pancreas and lung)	MEKi: Trametinib.	SHOC2, BCL2L1, MCL1, EXT1, EXT2, EXTL3, SLC35B2.
Han et al. *Nature.* 2020 [123]	Genome-Wide custom CRISPR library	2D vs. 3D Pooled CRISPR-Cas9 screen (proliferation assays)	H23 KRAS-mutant cells (KRAS^G12C^). Lung adenocarcinoma (LUAD)	KRASi: ARS-853	CPD, IGF1R
Michels et al. *Cell Stem Cell.* 2020 [124]	Custom CRISPR library (85 tumor suppressor genes)	In vivo screen (tumor growth study)	Colon organoids (APC^−/−^/KRAS^G12D^) vs. cancer cell lines	CRISPR-UMI validation screen (281 sgRNAs)	TGFBR2
Kelly et al. *Cancer Discov.* 2020 [125]	Custom CRISPR Double Knockout (CDKO) library (119 genes, 7021 pairs)	CDKO screen for genetic interactions (proliferation assays)	2 KRAS-mutant cell lines (A549/H23). LUAD	Focused CDKO screen in 9 LUAD cell lines	RHOA-RAP1GDS1 combination
Li et al. *Cancer Discov.* 2020 [126,127]	Custom CRISPR library (524 epigenetic regulators)	Epigenetic-focused CRISPR KO in vivo screen	KP mutant lung cancer mouse model	Drugs: anti-PD1 or isotype control	Asf1a, Npm1
Takahashi et al. *Mol cell.* 2020 [128]	Custom CRISPR-Cas9 library (1500 NRF2-hyperactivated related genes)	2D vs. 3D Pooled CRISPR-Cas9 screen (proliferation assays)	A549/H1437 LUAD 2D cell lines and 3D spheroids	N/A	TSC1, GPX4
Wei et al. *Proc Natl Acad Sci USA.* 2020 [129]	CRISPR library from Dr. Sabatini (619 human genes)	Pooled CRISPR knockout sensitizer screen	PDX366 model (KRAS, P53 and SMAD4 mutant). Pancreatic PDX-cells	Drug: Gemcitabine	PRMT5
Jung et al. *Oncogene.* 2021 [130]	Genome-Wide CRISPR/Cas9 library	Pooled CRISPR knockout sensitizer screen	SW620 cells (KRAS^G12V^) and HCT116 cells (KRAS^G13D^). Colorectal cancer	Drug: ABT-263	WNT signaling pathway; BCL-2 family genes
Biancur et al. *Cell Metab.* 2021 [131]	Custom CRISPR/Cas9 KO library (3000 mouse metabolic genes)	Pooled CRISPR KO screen: in vitro and in vivo (proliferation/viability assays)	C57BL/6 mouse PDA cell line (KRAS^G12D^)	Additional CRISPR screen in a 3D culture model	Fdft1; cholesterol synthesis
Yu et al. *Oncogene.* 2022 [132]	Genome-Wide CRISPR/Cas9library (human GeCKO)	Pooled CRISPR knockout sensitizer screen	HCT116 cells (KRAS^G13D^). Colorectal cancer	MEKi: AZD6244	GRB7; RTK pathway

## Data Availability

Not applicable.
